# On Applicability of Quantum Formalism to Model Decision Making: Can Cognitive Signaling Be Compatible with Quantum Theory?

**DOI:** 10.3390/e24111592

**Published:** 2022-11-02

**Authors:** Andrei Khrennikov

**Affiliations:** International Center for Mathematical Modeling in Physics and Cognitive Sciences, Linnaeus University, SE-351 95 Växjö, Sweden; andrei.khrennikov@lnu.se

**Keywords:** Bell inequality, signaling, marginal inconsistency, nonlocality, contextuality, incompatibility, quantum-like modeling, decision making, cognition, quantum measurement theory, Born rule, subjective probability, QBism

## Abstract

This note is devoted to the problem of signaling (marginal inconsistency) in the Bell-type experiments with physical and cognitive systems. It seems that in quantum physics, this problem is still not taken seriously. Only recently have experimenters started to check the signaling hypothesis for their data. For cognitive systems, signaling was statistically significant in all experiments (typically for decision making) performed up to today. Here, one cannot simply ignore this problem. Since signaling contradicts the quantum theory of measurement for compatible observables, its statistical significance in experiments with humans can be considered as an objection for quantum-like modeling—applications of quantum theory to cognition, decision making, psychology, economics and finance, social and political science. In this paper, we point to two possible sources of signaling generation that are consistent with quantum measurement theory. Thus, the signaling objection for quantum-like modeling is not catastrophic. One of these sources is the direct physical signaling about selection of experimental settings, questions or tasks in quantum-like studies. Another possible source is a state modification dependent on experimental settings. The latter was a rather common source of signaling in quantum physics. Since the physical size of the brain is very small comparing with the light velocity, it seems to be impossible to prevent the direct physical signaling (with electromagnetic waves) between the brain’s areas processing two questions *a* and b. However, if, for these questions, not the electromagnetic waves, but electrochemical communication plays the crucial role, the experimenter may hope to make signaling weaker by answering the questions faster. The problem of question-dependent mental state modification seems to be solvable via smarter experimental design. This paper can be useful both for physicists interested in quantum foundations and for researchers working in quantum-like studies, e.g., applying the quantum theory to model decision making or psychological effects. This paper is solely about quantum theory. Thus, we do not consider general contextual probabilistic models.

## 1. Introduction

The Bell inequalities [1,2,3,4] are the basic tools to compare the probabilistic structures of classical and quantum physics, both theoretically and experimentally. It is impossible to create a representative sample from the colossal ensemble of publications in this area. The author points only to publications that he has read or himself contributed to their writing (see [5,6,7,8,9,10,11,12,13,14,15,16,17,18,19,20,21,22,23,24,25,26,27,28,29,30,31,32,33,34,35,36,37,38,39,40,41,42,43,44,45,46,47,48,49,50,51,52,53,54,55,56,57]). See Section 6 for the discussion on the interpretations for the violation of the Bell inequalities.

The aim of this paper is to discuss the problem of signaling (marginal inconsistency), which is often ignored in theoretical and experimental research on the Bell inequalities. (See Section 2 for the mathematical definitions.) Moreover, we plan to concentrate on quantum observables and quantum formalism, i.e., we are not interested in studies based on generalized probabilistic theories. Our final aim is to consider the problem of signaling in quantum-like modeling (see below).

Consider observables a,b,c. The marginal inconsistency for an observable *a* is the inconsistency of the probability distributions for pairs of observables (a,b) and (a,c). Their marginal probability distributions with regard to *b* and *c* are not equal. Typically, a physicist imagines that this mismatching of marginals is a consequence of the presence of real physical signaling between the measurement apparatuses for *a* and b. This is the origin of the rather ambiguous term “signaling”, which is commonly used in physics.

In this paper, the notion of observable is used in three senses:

- (a) An “experimental observable”; in physics, it is based on some measurement apparatus, in quantum-like applications, it is a question, task, or a problem for decision making;
- (b) A random variable representing an “experimental observable” in the classical (Kolmogorov) probability model;
- (c) A Hermitian operator (or more generally POVM) representing an “experimental observable” in the quantum probability model.

In the (a)-case, probability is the “experimental probability”, i.e., the frequency of the registration of the concrete outcome of measurements; in the (b)-case, probability is defined as a probability measure; in the (c)-case it is given by the Born rule. The experimental probabilities can be considered as the approximations of classical and quantum probabilities. For groups of compatible “experimental observables”, i.e., jointly measurable, classical and quantum probabilistic descriptions are equivalent. Therefore, we would not specify what kind of probability is considered. We hope that this framework would not disturb the reader too much. Otherwise, we should use say the symbols $a,\xi_{a},\hat{a}$ for physical observable, the corresponding random variable and Hermitian operator as well as the symbols $P_{\exp},\;P_{\mathrm{class}},\;P_{\mathrm{quant}}$ for experimental probability-frequency, the corresponding classical probability measure and quantum probability given by Born’s rule.

As was emphasized in the review [58], signaling patters can be found in practically all statistical datasets collected in the Bell experiments. This is natural. Signaling can be generated by noise and some technicalities in experimental setting. However, signaling for joint measurements is forbidden by quantum theory (see Section 3.1). We are sure that by making experiments cleaner, experimenters can make the signaling degree essentially lower. So, even statistically significant signaling in data as, for example, in experiments [12,13,14,15,19] is not considered as a sign that the laws of quantum mechanics are violated. In the loophole free experiments [20,21] signaling is statistically insignificant. At the same type, the first experiment claiming that all basic loopholes were closed [19] suffered from statistically significant signaling [22].

It should be pointed out that initially the problem of signaling in the Bell experiments was practically ignored. The interest to this topic was stimulated by the works of Adenier and author [22,59,60,61,62]. The important role was played by involvement of Weihs into debate on signaling in experimental data sets. First, he sent to us the rough (click by click) data from his experiment, which closed the locality loophole [13] (see also [14]). Then, he then openly recognized the presence of signaling and proposed some explanation of its generation [63]. This explanation will be discussed in the present paper and coupled to quantum-like modeling (Section 3.3).

Recently, the methodology and the formalism of quantum theory started to be widely used outside of physics, in cognition, psychology, decision making, economics and finance, AI, social and political science (see the monographs [64,65,66,67,68,69,70,71,72] and the recent reviews [73,74]). This area of science is known as *quantum-like modeling*. In particular, following to quantum theory researchers explored the Bell tests, which were interpreted as the tests of non-classicality and contextuality [75,76,77,78,79,80,81,82,83].

Since all interpretational problems of quantum physics are automatically projected on quantum-like theory, the violation of the Bell inequalities for experiments with humans is also characterized by the diversity of interpretations (Section 6). *The author advertizes the incompatibility interpretation* [46,47,48,49,50,51,52,53] (see also [83]). The latter means that generally outcomes of mental observables, say questions asked to people, cannot be treated as predetermined. They are created in the process of, e.g., answering to questions. The mental observables are not (self-)measurements of the objective properties of the brain, which is understood as an information processor. Hence, the Bohr complementarity principle is also the fundamental principle of quantum-like modeling. We remark that Bohr emphasized that the domain of applicability of this principle is not restricted to physics, but it also includes biology [84]. We also recall that Bohr borrowed the complementarity principle from psychology—from James [85] (see also [72] for a discussion). Thus, we can say that exploring of the complementarity principle in cognitive studies, decision making, and psychology is its comeback powered by the mathematical formalism of quantum theory.

Surprisingly, all experimental statistical data collected in quantum-like studies demonstrated statistically significant signaling (this was pointed out in [78]). In principle, one could guess that signaling in such data is not irreducible and its disappearance is only the matter of more careful experimenting. The data presented in article [79] may support such expectation on elimination of signaling from data in experiments with humans. However, the samples collected in this article were relatively small, and the absence of signaling for some experimental contexts might be a consequence of insufficiency of the sample size.

Generally, the degree of the belief in applicability of quantum theory in aforementioned areas of research is essentially lower than in physics. (We remark that even in physics, the applicability of quantum theory is only the matter of belief based on its successful use up to today. In contrast to, e.g., relativity theory, quantum theory is not based on some heuristically clear principles, as say constancy of the light velocity. See, e.g., Zeilinger’s paper [86] for the corresponding discussion.) *One may speculate that strong signaling in data collected in decision making experiments with humans is a sign of inapplicability of quantum theory.*

We remark that another objection to quantum-like modeling was presented in article [87] by proving that some combinations of psychological effects cannot be described by the von Neumann measurement theory. The latter is based on the projection type state update. This problem was resolved by considering more general quantum observables within theory of quantum instruments [88,89].

The doubts in applicability of quantum theory to mental phenomena were strengthen by article [82]. In the experiments presented in this paper the CHSH -correlations essentially exceeded the Tsirelson bound. If it would be possible to eliminate signaling completely, the CHSH-correlations would approach the value four.

In this note, we show that such a situation does not contradict quantum theory: both signaling and overcoming of the the Tsirelson bound can be described by the quantum formalism. Thus, one can continue to use quantum-like models, e.g., in decision making. We point to two possible sources of signaling compatible with quantum theory.

One of them is going back to Weihs’ paper [63], where he related the appearance of the signaling pattern in his statistical data with state variability. In fact, the latter also led to signaling in another breakthrough experiment [15] in which the detection loophole was closed (see Section 3.3). It seems that such a source of signaling can be eliminated even in the decision making experiments. So, this paper can stimulate researchers who plan to test the Bell inequalities with experiments with humans. They can try to find the ways to exclude the sources of the mental state variablity.

Another source is the direct signaling in the form of communication on experimental settings—in cognitive studies, these are questions asked to a person. This may be physical signaling, e.g., via electromagnetic waves. They propagate between the areas of the brain processing two questions, *a* and *b*, which were asked to a person. Since the brain size is very small compared to the light velocity, such signaling cannot be eliminated (so it seems that cognitive experiments similar to Weih’s experiment [14,63] are impossible). For readers who are far from quantum-like modeling, say physicists, or biologists, or sociologists, the scheme of quantum-like formalization of the process of decision making is presented in Section 8.

## 2. Signaling—Marginal Inconsistency

Consider observables a,b,c of any sort. Suppose that the joint pairwise measurements of (a,b) and (a,c) are possible, i.e., the joint probability distributions P(a=α,b=β) and P(a=α,c=γ) are well defined. For simplicity, we suppose that the ranges of values of these observables are discrete sets, $X_{a}=\left(\alpha_{j}\right),X_{b}=\left(\beta_{j}\right),X_{c}=\left(\gamma_{j}\right)$. It is natural to assume that the probability distributions for measurements of each of these observables are well defined: P(a=α),P(b=β),P(c=γ).

If these observables can be described by classical probability theory, then the condition of marginal consistency holds, e.g.,

(1) $$P\left(a=\alpha \right)=\sum_{\beta \in X_{b}}P\left(a=\alpha ,b=\beta \right),\;P\left(a=\alpha \right)=\sum_{\gamma }P\left(a=\alpha ,c=\gamma \right),$$

and, hence,

(2) $$\sum_{\beta \in X_{b}}P\left(a=\alpha ,b=\beta \right)=\sum_{\gamma \in X_{c}}P\left(a=\alpha ,c=\gamma \right),$$

the latter is called the condition of no-signaling: selection of a co-measurable observable does not change the probability distribution for *a* measured alone. The same conditions can be considered for other observables.

If observables are not described by classical probability theory, then in principle condition (2) can be violated, this is the situation of marginal inconsistency. In physical literature, this situation is known as signaling. May be the latter terminology is ambiguous, but it is commonly used in physical papers.

## 3. Sources of Signaling Compatible with Quantum Formalism

As is predicted by quantum theory, in the joint measurements of compatible quantum observables marginals (giving the probabilities of the outcomes for each observable) are consistent with JPDs obtained in the pairwise measurements. This simple fact can be easily proven. We repeat once again that we are interested in applicability of quantum theory, in applicability of the complex Hilbert space formalism. So, we do not discuss generalized probabilistic theories.

### 3.1. Marginal Consistency (No Signaling) for Compatible Quantum Observables

Consider a state given by density operator ρ and three quantum observables a,b,c represented by the Hermitian operators A,B,C (acting in a complex Hilbert space H). For simplicity, we assume that these operators have the purely discrete spectra $X_{a}=\left(\alpha_{j}\right),X_{b}=\left(\beta_{j}\right),X_{c}=\left(\gamma_{j}\right)$. Denote their spectral spectral families of projectors by $E^{a}\left(x\right),E^{b}\left(x\right),E^{c}\left(x\right),$ i.e.,

$$A=\sum_{\alpha \in X_{a}}\alpha E^{a}\left(\alpha \right),\;B=\sum_{\beta \in X_{b}}\beta E^{b}\left(\beta \right),\;C=\sum_{\gamma \in X_{c}}\gamma E^{c}(\gamma ).$$

It is assumed that in each pair (a,b) and (a,c) the observables are compatible, mathematically this means that

[A,B]=0,[A,C]=0.

Generally observables *b* and *c* can be incompatible, in such a case [B,C]≠0.

Consider a quantum state given by density operator ρ. By the Born rule, the probabilities for measurements of observables and the joint measurements for pairs (a,b) and (b,c) can be represented as follows:

(3) $$P\left(a=\alpha |\rho \right)=\mathrm{Tr}\rho E^{a}\left(\alpha \right),$$

(4) $$P\left(b=\beta |\rho \right)=\mathrm{Tr}\rho E^{b}\left(\beta \right),$$

(5) $$P\left(c=\gamma |\rho \right)=\mathrm{Tr}\rho E^{c}\left(\gamma \right),$$

and

(6) $$P\left(a=\alpha ,b=\beta |\rho \right)=\mathrm{Tr}\rho E^{a}\left(\alpha \right)E^{b}\left(\beta \right),$$

(7) $$P\left(a=\alpha ,c=\gamma |\rho \right)=\mathrm{Tr}\rho E^{a}\left(\alpha \right)E^{c}(\gamma ).$$

Hence

(8) $$\sum_{\beta \in X_{b}}P\left(a=\alpha ,b=\beta |\rho \right)=\mathrm{Tr}\rho E^{a}\left(\alpha \right)\sum_{\beta \in X_{b}}E^{b}\left(\beta \right)=\mathrm{Tr}\rho E^{a}\left(\alpha \right)$$

and

(9) $$\sum_{\gamma \in X_{c}}P\left(a=\alpha ,c=\gamma |\rho \right)=\mathrm{Tr}\rho E^{a}\left(\alpha \right)\sum_{\gamma \in X_{c}}E^{c}\left(\gamma \right)=.\mathrm{Tr}\rho E^{a}(\alpha ).$$

### 3.2. Signaling on Selection of Experimental Settings

Consider the scheme of the Bell experiment: a source generating the pairs of systems $S=(S_{1},S_{2})$ and two measurement devices, in Alice’s and Bob’s labs, with dichotomous “yes-no” outputs. Denote settings of measurement devices as θ and ϕ, respectively. In quantum optics, these are angles of orientations of polarization beam splitters. However, we consider more general situation with an attitude to clarify signaling in experiments with cognitive systems in the process of decision making.

Suppose now that the quantum observables representing measurements on $S_{1}$ and $S_{2}$ depend on both orientations,

(10) a=a(θ,ϕ),b=b(θ,ϕ).

They are represented by operators

(11) A=A(θ,ϕ),B=B(θ,ϕ).

Thus, selection of setting ϕ for PBS in Bob’s lab changes the observable (measurement procedure) in Alice’s lab and vice versa.

Such nonlocal dependence can be generated by signaling between Bob’s lab and Alice’s lab carrying information about experimental setting selections.

This situation can also be referred to as the absence of free will of experimenters with regard to selection of experimental settings. However, we would not follow this line of thought, which is so natural for the philosophy of superdeterminism.

We are mainly interested in quantum-like applications where the parameters θ and ϕ have the meaning of the questions, which are jointly asked to some person participating, e.g., in a social opinion pool. In this framework, both “labs” are located in the same brain, and Alice-lab and Bob-lab can be in principle associated with different brain’s areas. However, communication between these areas includes the the electromagnetic waves signaling. Therefore, both brain’s areas are connected at the information level and by answering the θ-question, one takes into account the ϕ-question and vice versa.

In such a situation,

(12) $$P\left(a\left(\theta ,\phi \right)=\alpha ,b\left(\theta ,\phi \right)=\beta \right)=\mathrm{Tr}\rho E^{a(\theta ,\phi )}\left(\alpha \right)E^{b(\theta ,\phi )}\left(\beta \right)$$

and hence

(13) $$\sum_{\beta }P\left(a\left(\theta ,\phi \right)=\alpha ,b\left(\theta ,\phi \right)=\beta |\rho \right)=\mathrm{Tr}\rho E^{a(\theta ,\phi )}\left(\alpha \right)\sum_{\beta }E^{b(\theta ,\phi )}\left(\beta \right)$$

$$=\mathrm{Tr}\rho E^{a(\theta ,\phi )}\left(\alpha \right),$$

(14) $$\sum_{\beta }P\left(a\left(\theta ,\phi \right)=\alpha ,b\left(\theta ,\phi^{\prime }\right)=\beta |\rho \right)=\mathrm{Tr}\rho E^{a(\theta ,\phi^{\prime })}\left(\alpha \right)\sum_{\beta }E^{a(\theta ,\phi^{\prime })}\left(\beta \right)$$

$$=\mathrm{Tr}\rho E^{a(\theta ,\phi^{\prime })}(\alpha ).$$

Generally,

(15) $$\mathrm{Tr}\rho E^{a(\theta ,\phi )}\left(\alpha \right)\neq \mathrm{Tr}\rho E^{a(\theta ,\phi^{\prime })}\left(\alpha \right).$$

or, in the probabilistic terms,

(16) $$P\left(a\left(\theta ,\phi \right)=\alpha |\rho \right)\neq P\left(a\left(\theta ,\phi^{\prime }\right)=\alpha |\rho \right).$$

This mutual dependence may also lead to violation of the Bell inequalities. Moreover, the Tsirelson bound can be violated.

We remark that decomposition of *S* into subsystems $S_{1}$ and $S_{2}$ and association of observables *a* and *b* with these subsystems did not play any role in quantum calculations. Such decomposition and coupling it with spatial locality is important only in the physics as the sufficient condition to prevent signaling on selection of experimental settings.

In cognitive experiments, observables are typically questions asked to a system *S* (e.g., a human). As we have seen, the dependence of questions *a* and *b* on the same set of parameters can generate signaling. This dependence is not surprising. Even if questions *a* and *b* are processed by different regions of the brain, the physical signaling between these regions cannot be neglected. If θ and ϕ are the contents of the *a*- and *b*-questions, then after a few milliseconds, the area of the brain processing a=a(θ) would get to “know” about the content of the *b*-question, and thus *a*-processing would depend on both parameters: a=a(θ,ϕ). We remark that an essential part of information processing in the brain is performed via an electromagnetic field; such signals propagate with the light velocity, and the brain is very small as a physical body.

On the other hand, some kind of mental localization must be taken into account; mental functions performing different tasks use their own information resources (may be partially overlapping). Without such mental localization, the brain would not be able to discriminate different mental tasks and their outputs. At least for some mental tasks (e.g., questions), dependence of *a* on the parameter ϕ (see (10)) can be weak. For such observables, signaling can be minimized. (The real situation is more complex; not only the brain, but the whole nervous system is involved in mental processing.)

### 3.3. Signaling from State Dependence on Experimental Settings

Let us turn to quantum physics. Here, “signaling” often has the form of real physical signaling and it can reflect the real experimental situation. We now discuss the first Bell-experiment in which the detection loophole was closed [15]. It was performed in Vienna by Zelinger’s group, and it was characterized by statistically significant signaling. By being in Vienna directly after this experiment, the author of this paper spoke with people who did it.

They told the following story about the origin of signaling—marginal inconsistency. The photon source was based on the laser generating emission of the pairs of entangled photons from the crystal. It happened (and it was recognized only afterwards) that the polarization beam splitters (PBSs) reflected some photons backward, and by approaching the laser, they changed its functioning and backward flow of photons depended on the orientations of PBSs. In this situation, “signaling” was not from *b*-PBS to *a*-PBS, but both PBSs sent signals to the source. Selection of the concrete pair of PBSs changed functioning of the source; in the quantum terms, this means modification of the state preparation procedure. In this case, selection of a pair of orientations leads to the generation of a quantum state depending on this pair, $\rho_{ab}.$ This state modification contributed to the signaling pattern in the data.

The above physical experimental illustration pointed to the state’s dependence on the experimental context as a possible source of signaling. It is clear that, for $\rho =\rho_{a,b},$ generally

(17) $$\mathrm{Tr}\rho_{\mathrm{ab}}E^{a}\left(x\right)\neq \mathrm{Tr}\rho_{\mathrm{ac}}E^{a}(x).$$

This dependence also may lead to violation of the Bell inequalities. Moreover, the Tsirelson bound can be violated.

We remark that it seems that the state variability depending on experimental settings was the source of signaling in Weihs’ experiment [13], which closed the nonlocality loophole. At least in this way, we interpreted his reply [63] to our (me and Guillaume Adenier) paper [60]. Since Weihs [13] was able to separate two “labs” to a long distance, the signals from one lab could not approach another during the process of measurement.

In quantum physics, experimenters were able to block all possible sources of the state’s dependence on the experimental settings. Thus, it is claimed that one can be sure that ρ does not depend on *a* and b. By using the orientations of PBSs θ,ϕ, i.e., ρ=ρ(θ,ϕ), the latter condition can be written as

(18) $$\frac{\partial \rho (\theta ,\phi )}{\partial \theta }=0,\;\frac{\partial \rho (\theta ,\phi )}{\partial \phi }=0.$$

The stability of state preparation is the delicate issue. As we have seen, the source by itself can be stable and generate approximately the same state ρ, but the presence of measurement devices can modify its functioning. Moreover, even if any feedback to the source from measuring devices is excluded, the laser’s functioning can be disturbed by fluctuations. Typically, violation of state stability cannot be observed directly and the appearance of a signaling pattern can be considered as a sign on the state’s variation. In physics, the signaling can be rigidly associated with fluctuations in state preparation. Spatial separation leads to local parameter dependence of observables, i.e., a=a(θ) and b=b(ϕ).

## 4. Projection Valued Measures

We considered the model in which quantum observable *a* is represented by Hermitian operator A. For the purely discrete spectrum, its spectral decomposition has the form:

(19) $$A=\sum_{x\in X_{a}}x\;E^{a}\left(x\right),$$

where $E^{a}\left(x\right)$ is the orthogonal projector onto the subspace of H corresponding to the eigenvalue x, i.e., $H_{x}^{A}=E^{a}\left(x\right)H.$ We recall that the spectral family of orthogonal projectors satisfies the normalization condition

(20) $$\sum_{x}E^{a}\left(x\right)=I.$$

where *I* is the unit operator, and the mutual orthogonality condition

(21) $$E^{a}\left(x\right)⊥E^{a}\left(y\right),x\neq y.$$

Probability for the outcome *x* is given by the Born rule $P\left(a=x|\rho \right)=\mathrm{Tr}\rho E^{a}\left(x\right).$

In physics, the values of observables are real numbers, the eigenvalues of Hermitian operators. We point out that the right hand side of the Born rule contains only orthogonal projectors and the association of them with real numbers is not crucial, *x* is just an index labeling projectors. This label can belong to any set $X_{a}$ and then the probability distribution given by the Born rule is defined on the set $X_{a}.$ For example, *X* can be the set of all possible words in the Russian or Chinese language, or the set of possible conscious experiences, decisions, questions, and emotions. This possibility to operate with an arbitrary set of symbols $X_{a}$ is very important for applications outside of physics. For simplicity, we consider a finite set of outcomes:

(22) $$X_{a}=\left\{x_{1},\ldots ,x_{m}\right\}.$$

Now, let us represent an observable *a* (physical or mental) with the range of values $X_{a}$ by the family of orthogonal projectors, $E={\left(E^{a}\left(x\right)\right)}_{x\in X_{a}}$ satisfying two constraints (20), (21). They guarantee that, for any state ρ, the quantity determined by the Born rule can be interpreted as probability, i.e., it is normalized by one

(23) $$\sum_{x\in X_{a}}P\left(a=x|\rho \right)=1.$$

Such a family of projectors ${\left(E^{a}\left(x\right)\right)}_{x\in X}$ determines a *projector-valued measure* (PVM),

(24) $$\mu^{a}\left(G\right)=\sum_{x\in G}E^{a}\left(x\right),$$

where *G* is a subset of $X_{a}.$

We can use PVMs for mathematical representation of observables. This is a very natural generalization of operator-representation. It is clear that considerations of Section 3.1 are still valid for observables given by PVMs $\mu^{a},\mu^{b},\mu^{c}.$ Observables (a,b) and (a,c) are compatible if the corresponding PVMs commute.

## 5. Generalized Observables: Positive Operator Valued Measures

Generalized quantum observables are given by POVMs. We restrict considerations to POVMs with a discrete domain of definition $X=\{x_{1},\ldots ,x_{m}\}.$ POVM is a map x→Π(x).

Here, for each x∈X,Π(x) is a positive contractive self-adjoint operator (i.e., 0≤Π(x)≤I) (called an *effect*), and the normalization condition

(25) $$\sum_{x}\Pi \left(x\right)=I$$

holds, where *I* is the unit operator. This map defines an operator valued measure on algebra F of all subsets of set X. For O⊂X,

$$\Pi \left(O\right)=\sum_{x\in O}\Pi \left(x\right).$$

The condition (25) is the operator-measure counterpart of the condition normalization by 1 for usual probability measures.

We stress that the PVMs form a special class of POVM.

POVM Π represents statistics of measurements for an observable *a* with the following generalization of the Born rule:

P(a=x|ρ)=Tr[ρΠ(x)].

We remark that equality (25) implies that $\sum_{x}P\left(a=x|\rho \right)=1.$

*POVMs are generalized quantum observables*. This is the natural extension of the class the von Neumann observables given by PVMs.

Consider now two generalized observables *a* and *b* given by POVMs $\Pi^{a}\left(x\right),x\in X_{a},$ and $\Pi^{b}\left(y\right),y\in X_{b}.$ If the observables are compatible, then there exists POVM $\Pi^{a,b}\left(x,y\right),\left(x,y\right)\in X_{b}\times X_{a},$ such that

(26) $$\Pi^{a}\left(x\right)=\sum_{y\in X_{b}}\Pi^{a,b}\left(x,y\right),\;\Pi^{b}\left(y\right)=\sum_{x\in X_{a}}\Pi^{a,b}\left(x,y\right).$$

In fact, these equalities may be treated as the operator version of the marginal consistency condition (1).

Now, if the observables in each pair (a,b) and (a,c) are compatible, then there exist two POVMs $\Pi^{a,b}\left(x,y\right),\left(x,y\right)\in X_{b}\times X_{a},$ and $\Pi^{a,c}\left(x,z\right),\left(x,z\right)\in X_{a}\times X_{c},$ and (26) is completed by

(27) $$\Pi^{a}\left(x\right)=\sum_{z\in X_{c}}\Pi^{a,c}\left(x,z\right),\;\Pi^{c}\left(y\right)=\sum_{x\in X_{a}}\Pi^{a,c}\left(x,z\right).$$

Born’s rule for generalized compatible observables has the form:

$$P\left(a=x,b=y|\rho \right)=\mathrm{Tr}[\rho \Pi^{a,b}\left(x,y\right)],$$

$$P\left(a=x,c=z|\rho \right)=\mathrm{Tr}[\rho \Pi^{a,c}\left(x,z\right)].$$

Hence,

$$\sum_{y\in X_{b}}P\left(a=x,b=y|\rho \right)=\mathrm{Tr}\left[\rho \sum_{y\in X_{b}}\Pi^{a,b}\left(x,y\right)\right]=\mathrm{Tr}\left[\rho \Pi^{a}\left(x\right)\right],$$

$$\sum_{z\in X_{c}}P\left(a=x,c=z|\rho \right)=\mathrm{Tr}\left[\rho \sum_{z\in X_{c}}\Pi^{a,b}\left(x,y\right)\right]=\mathrm{Tr}\left[\rho \Pi^{a}\left(x\right)\right],$$

and thus

$$\sum_{y\in X_{b}}P\left(a=x,b=y|\rho \right)=\sum_{z\in X_{c}}P\left(a=x,c=z|\rho \right)=P\left(a=x|\rho \right).$$

Even generalized quantum observables satisfy the condition of marginal consistency.

## 6. Interpretations for the Violation of the Bell Inequalities

Although mathematically the Bell project is well justified, interpretations of the violation of the Bell inequalities are characterized by diversity and the intensive debates still continue. We are not able to mention all possible viewpoints. And this paper is not directed to such foundational issues. We mention just the nonlocal interpretation [1,2,3,4,9] (and one must differ Einsteinian and Bell nonlocalities [90,91,92,93]), the contextual interpretation [2,8,11,94,95,96,97,98,99,100,101,102,103,104,105,106,107,108,109,110,111,112,113,114], and the observables incompatibility interpretation [46,47,48,49,50,51,52,53,115,116].

We can also mention the *“probabilistic opposition”*; the members of this group claim that quantum nonlocality is apparent. It is a consequence of the misuse of probability theory. The probabilistic argumentation is diverse, as examples see, e.g., [23,24,25,26,27,29,30,31,32,33,34,35,36,37,38,42,54,55,56,57,91,93]. In fact, the probabilistic opposition, at least the majority of its members point out to the dependence of probabilities on the selection of experimental contexts. It is interesting that this dependence can be modeled even within the classical probability theory by treating the quantum probabilities as classical conditional probabilities [54,55,56,57] (see also Koopman [117] and Ballentine [118,119,120,121]).

From the purely mathematical side, the probability opposition is supported by the works of Boole [122,123] who considered the problem of the existence of the joint probability distribution for data collected in a few experiments. Later, Vorob’ev [124] resolved this problem in the most general setting and derived all possible inequalities, which nowadays are known as the Bell inequalities. Unfortunately, the works of Vorob’jev were ignored by the probability community, which was rigidly structured within the classical probability theory based on the Kolmogorov axiomatics (1933) [125]. Quantum physics played the crucial stimulating role in destruction of this classical probabilistic monolith, and Bell’s contribution has to be highly estimated, in spite of his addiction to the nonlocal interpretation. By proving his famous theorem [10], Fine turned the studies on the violation of the Bell inequalities to the Boole–Vorobj’ev pathway. We should also remember the contribution of Pitowsky, who “rediscovered” Boole’s works [126,127]. The works of Vorobj’ev were “rediscovered” by Philipp [128], who advertised them during the Växjö conference series on quantum foundations.

This is a good place to remark that experimenters typically use the nonlocal interpretation and interpret the nonlocality as Einsteinian nonlocality—the existence of spooky action at a distance. This is the output of my conversations with top experimenters dealing with the Bell inequalities. The exception from this rule are experimenters dealing with contextuality tests [17,18]. We also remark that Bell by himself pointed to the contextual interpretation in one of the first papers [2], see also Gudder [6,7] (and Shimony [8]). At the beginning, the term contextuality was not in use. It was invented in the paper of Beltrametti and Cassinelli [129].

Over many years, the author was one of the active members of the probabilistic opposition. He wrote numerous works explaining the violation of the Bell inequalities by contextuality of probabilities (see, e.g., monographs [130,131,132]. However, recently, the author became more concentrated on finding the quantum mechanical (QM) explanation of the origin of contextuality and, as was shown in articles [46,47,48,49,50,51,52,53] (see also Jaeger [115,116]), the seed of contextuality is in the existence of *incompatible observables.* From this viewpoint, the violation of the Bell inequalities can be explained by *the Bohr complementarity principle*—one of the fundamental principles of QM.

## 7. Concluding Remarks

We hope that this paper will stimulate interest for the problem of signaling (marginal inconsistency) in data collected in the Bell-type experiments. This paper can be useful both for quantum and quantum-like studies. In quantum physics, the signaling problem is still not highlighted so much. Experimental studies often miss checking whether signaling in data is statistically insignificant or not. In quantum-like studies, the signaling problem cannot be ignored, and the problem of the explanation of signaling within quantum theory arose.

The straightforward application of the quantum formalism for joint measurements of compatible observables (in fact, the Born rule) implies no signaling. Hence, applicability of this formalism to the mathematical description of the results of the Bell-type tests with humans can be questioned.

We remark that signaling can be easily modeled within generalized probability theories (such as the Växjö contextual probability model [130,131,132] or Contextuality by Default [110,111,112,113,114]). However, we are interested not in the general problem of mathematical modeling of, say, cognition and decision making, but in the possibility to use the formalism of quantum theory: representation of mental states by density operators and questions (tasks) by Hermitian operators and the use of the Born rule for calculation of outcomes’ probabilities. Therefore, we searched for possibilities to describe signaling within quantum theory.

It was shown that, in principle, it is possible. We also discussed whether such signaling can be eliminated in experiments with humans. Maybe this paper would be of some use for researchers planning new Bell-type experiments with humans.

For physicists, we remark that it seems that in experiments on quantum contextuality, i.e., measurement of compatible observables on a single system, say a neutron, the signaling hypothesis has never been checked. We cannot exclude that statistical datasets from such experiments contain statistically significant signaling patters. If signaling would be found in such data, it might help to understand signaling in decision making experiments with humans. Such experiments testing contextuality in quantum and quantum-like studies are similar in the sense that these are single system experiments, one neutron (or atom) and one human.

And finally we remark that the foundational studies in quantum physics suffer from the impossibility to work with rough (click-by-click) data from the Bell-type (and other foundationally important) experiments. It would be natural to put such data on the web-page created for each experiment or create a single database for at least most experiments. The author and Adenier were able to receive data only from three experiments [12,13,19]. All these data-sets demonstrated statistically significant signaling. The author even wrote a kind of pamphlet “Unuploaded experiments have no result” [133]. The discussion on the necessity of such a database has been going during the last ten years, but without any result. It also often happened that data appeared after an experiment, but then after a few months it disappeared from the Internet.

## 8. Appendix: Quantum-like Modeling of Decision Making

Here, we briefly present the general quantum-like scheme for decision making (see, e.g., [64,65,66,67,68,69,70,71,72,73,74] for details and variations).

### 8.1. Quantum-like Representation of Belief (Mental) State

The belief (mental) state of a decision maker, say Alice, is represented as a quantum state, typically by a pure state ψ belonging to a complex Hilbert space H (space of belief states). Generally, a belief state is a mixed state represented by a density operator ρ. (A density operator ρ is determined by conditions: $\rho =\rho^{⋆},\rho \geq 0,\mathrm{Tr}\rho =1.)$ In this Appendix, for simplicity, we typically restrict considerations to pure states (besides Section 8.2.2).

The problem of the belief state’s interpretation is the cognitive counterpart of the problem of the quantum state interpretation—the “wave function interpretation problem”. The latter is one the main foundational problems of QM, and it is characterized by the diversity of the viewpoints. Some experts consider this interpretational diversity as a sign of the deep crises in quantum foundations. Other experts think that such diversity is not a problem—“one can use an interpretation which he likes!” However, the majority of quantum physicists do not even think about the state interpretation problem. They formally operate within the quantum formalism. They are disappointed by the question: “ What state’s interpretation do you use?” Often, one automatically replies: “I use the Copenhagen interpretation of QM!” However, an attempt to clarify the meaning of this statement may lead even to more disappointment. In fact, the latter is not surprising, because the Copenhagen interpretation is also characterized by the diversity of interpretations. Plotnitsky even suggested to speak about the interpretations in the spirit of Copenhagen, e.g., [134,135]. This interpretational ambiguity is projected to quantum-like modeling. However, the majority of researchers working with applications of the quantum formalism outside of physics proceed formally, without even trying to think about the interpretation problem for the quantum-like belief-state. We shall discuss the belief state’s interpretation in Section 9.

In quantum-like modeling of decision making, one typically considers only finite dimensional state spaces, as typically one also does in quantum information theory. We remark that real physics is based on infinite dimensional Hilbert spaces, such as the space $L_{2}$ of square integrable functions.

By choosing in H some orthonormal basis, H can be represented as the space of vectors with complex coordinates, $\psi =\left(z_{1},z_{2},\ldots ,z_{n}\right),z_{j}\in C,$ where C is the set of complex numbers. The scalar product is defined as

(28) $$\langle u|v\rangle =\sum_{i}{\bar{u}}_{i}v_{i},u=\left(u_{1},\ldots ,u_{n}\right),v=\left(v_{1},\ldots ,v_{n}\right),$$

where, for a complex number z=x+iy,x,y∈R, its conjugate is denoted by $\bar{z},$ here $\bar{z}=x-iy.$ The absolute value of *z* is given by ${\left|z\right|}^{2}=z\bar{z}=x^{2}+y^{2}.$

A pure quantum state is given as normalized vector ψ, i.e., ∥ψ∥=1, where vector’s norm is defined as

$${∥\psi ∥}^{2}=\langle \psi ,\psi \rangle .$$

### 8.2. Decisions as Quantum Observables

A decision problem is a question *a*, which is asked to Alice or a task which she must perform (to select some output). In the quantum-like framework, *a* is mathematically described by a quantum observable.

#### 8.2.1. Representation of Questions and Tasks as Quantum Observables—By Hermitian Operators, PVMs, or POVMs

In the von Neumann theory of quantum measurements, an observable *a* is mathematically represented by a Hermitian operator *A* (thus, $A=A^{⋆}).$ Its eigenvalues encode the possible outcomes, $\alpha_{1},\ldots ,\alpha_{k}.$ In decision making, these are answers to a question, a task, or possible solutions of a problem.

By choosing an orthonormal basis the space of belief states H can be represented as $H=C^{n}$ and an observable as a Hermitian matrix, i.e., $A=(a_{ij}),$ where

$$a_{ij}={\bar{a}}_{ji}.$$

Matrix’s eigenvalues encode the outputs of observations. The majority of problems of quantum-like modeling are coupled to dichotomous observables, i.e., $\alpha_{j}=0,1$ (or $\alpha_{j}=\pm 1).$ Thus, the quantum measurement model is mathematically formalized as the linear algebraic calculus for eigenvalues and matrices.

As was noted, in decision making and generally in quantum like modeling, it is not natural to solely use the Hermitian operator representation of the mental observables. This representation induces coupling of labels encoding possible answers or tasks with operator’s spectrum—a subset of R. It is more natural to operate with PVMs or POVMs.

#### 8.2.2. Quantum Dynamical Decision Making

In monograph [68] (see also [136]), we considered another quantum-like framework, which is know as *quantum dynamical decision making.* Here, the decision states are *steady states* of the open quantum system dynamics describing the belief (mental) state’s evolution in the process of decision making. Such modeling of generation of the decision states found many applications, from genetics and molecular biology to psychology and cognition, ecology and sociology.

In this approach, the belief state is mathematically described by a density operator or “mixed quantum state” and its evolution in the process of decision making, t→ρ(t), by the open quantum system dynamics. We emphasize that in this model, it is impossible to proceed solely with the pure states, the open quantum system dynamics immediately transfers a pure state given by a vector ψ into mixture of such states given by a density operator ρ. This is the result of interaction of the decision maker with the surrounding psycho-physical environment. A decision state is given by a *steady state*:

(29) $$\rho_{\mathrm{decision}}=\lim_{t\to \infty }\rho \left(t\right)$$

(if this limit exists). Of course, this limit procedure is just the mathematical idealization, it encodes damping of the state’s fluctuations, and damping is due to the interaction with the system’s environment. Hence,

(30) $$\rho_{\mathrm{decision}}\approx \rho \left(\tau \right),$$

for sufficiently large τ (w.r.t. decision maker’s temporal scale).

### 8.3. Probability of Decision

Consider the simplest mathematical representation of decisions, by Herminian operators. For Alice in the state ψ, the probability of decision $\alpha_{j}$ is given by the Born rule.

### 8.4. Transformation of Belief State Resulting from Decision Making

In the modeling of decision making, we are interested not only in the probabilities of various decisions, but also in transformation of belief states resulting from decision making. In the simplest model (due to von Neumann and Lüders), the decision with the outcome $a=\alpha_{j}$ induces projection of the belief state ψ (pure one) onto the $\alpha_{j}$-eigenspace of the Hermitian operator *A* describing, say, some question a.

However, this simple operation has only the restricted domain of applications. In contrast to physics, where one can proceed rather far with Hermitian operators and the state update of the projection type, in psychology and cognition, even the simplest psychological effects cannot be modeled in this way, and more general belief state updates have to be used, updates described by quantum instruments [88,89].

In open quantum system modeling of the process of decision making, the belief state is updated through approaching a steady state.

## 9. Interpretations of Belief State and Probability

The probability in the Born rule can be interpreted either statistically or subjectively. However, in QM, the interpretation problem is more complicated than in theories based on classical probability, say thermodynamics, because QM operates not solely with probabilities, but also with quantum states. Hence, not only probabilities, but also states have to be interpreted and the state-probability interpretation should be consistent.

### 9.1. Statistical Interpretation of State and Probability

By the statistical interpretation of probability, for a large ensemble of decision makers (*N* persons), the frequency of observation of the output a=α approaches (for N→∞) the probability calculated theoretically with the Born rule:

(31) $$\nu_{N}\left(\alpha \right)=n_{N}\left(\alpha \right)/N\approx {∥E\left(\alpha \right)\psi ∥}^{2},$$

where $n_{N}\left(\alpha \right)$ is the number of decision makers who answered a=α. This interpretation is the most useful to couple theory and experiment. In cognitive, psychological, social, and financial experiments statistical data is collected for an ensemble of humans (or animals).

In quantum physics, this interpretation of probability is coupled to the statistical interpretation of the wave function by which it represents not the state of an individual quantum system, but of an ensemble of systems prepared by the same preparation procedure. This interpretation is typically associated with Einstein, and later it was highlighted by Margenau and Ballentine. In decision theory, by the statistical interpretation, the belief state is associated with a large ensemble of decision makers, which is selected under special conditions—a decision making preparation procedure.

### 9.2. Individual Interpretation of State and Statistical Interpretation of Probability

Bohr and von Neumann assigned the wave function to an individual quantum system. However, for probability, they used the statistical interpretation. This interplay of individual (for state) and statistical (for probability associated with this state by the Born rule) interpretations made the statistical structure of QM really mystical. This mystery would be resolved only via the solution of the quantum measurement problem: creation of a mathematical model for generation of the concrete outputs of the observable *A* on the basis of the initial state ψ. This is the complicated problem and maybe it could not be solved at all within quantum theory. Nevertheless, the individual interpretation of the quantum state combined with the statistical interpretation probability is widely used in QM. It is reasonable to use this framework even in cognition, decision making, and other applications outside of physics.

By this interpretation, the belief (mental) state ψ is assigned to the individual decision maker, but it determines the statistical probability for an ensemble of decision makers who have the same state ψ. Why does ψ generate the statistical probability, which is experimentally verifiable (see (31)) with the frequency of observations $\nu_{N}\left(\alpha_{j}\right)?$ This question would be replied if, within quantum-like modeling, one would solve the measurement problem:

*How does the brain generate the concrete output $a=x_{j}$ starting with the mental state ψ?*

### 9.3. Subjective Interpretation of Probability and QBism

By the subjective interpretation of probability, Alice (whose belief state is ψ) assigns her own weight P(a=α) to the outcome a=α and in the quantum-like model of decision making this subjective probability is given by the Born rule, i.e., P(a=α)=P(a=α|ψ).

In general decision theory (i.e., not directly related to quantum experiments), the subjective interpretation of probability is widely applied to a variety of problems, including the framework of the subjective utility function. In the quantum-like reformulation, the Born rule gives subjective probabilities, which decision makers assign to possible outcomes.

In quantum physics, the subjective interpretation of probability is associated with *Quantum Baeysianism* (QBism). We start with the following citation of Fuchs and Schack [137], pp. 3–4:

*“The fundamental primitive of QBism is the concept of experience. According to QBism, quantum mechanics is a theory that any agent can use to evaluate her expectations for the content of her personal experience.*

*QBism adopts the personalist Bayesian probability theory… This means that QBism interprets all probabilities, in particular those that occur in quantum mechanics, as an agent’s personal, subjective degrees of belief. This includes the case of certainty - even probabilities 0 or 1 are degrees of belief…*

*In QBism, a measurement is an action an agent takes to elicit an experience. The measurement outcome is the experience so elicited. The measurement outcome is thus personal to the agent who takes the measurement action. In this sense, quantum mechanics, like probability theory, is a single user theory. A measurement does not reveal a pre-existing value. Rather, the measurement outcome is created in the measurement action.*

*According to QBism, quantum mechanics can be applied to any physical system. QBism treats all physical systems in the same way, including atoms, beam splitters, Stern-Gerlach magnets, preparation devices, measurement apparatuses, all the way to living beings and other agents. In this, QBism differs crucially from various versions of the Copenhagen interpretation…*

*An agent’s beliefs and experiences are necessarily local to that agent. This implies that the question of nonlocality simply does not arise in QBism.”*

See also the recent article of Fuchs [138].

What is about the QBism-interpretation of the belief state? (See [139] for the details.)

This is the theory about structuring the individual’s experiences in the situation of uncertainty. The belief state ψ is individual’s state. So, in physics, ψ is not the state of, say, an electron involved in the process of measurement, but of an experimenter who creates the subjective probabilistic picture for possible outcomes of this experiment.

However, QBism diminishes the role of the quantum state. QBists suggest operating solely with probabilities. To realize this approach, they consider non-standard quantum observables, which are mathematically represented by Hermitian operators, but generalized observables, which completely determine a quantum state. Such observables have $n^{2}$ outcomes, where *n* is the state space dimension. Another property is that the outcomes are not mutually exclusive, i.e., projections corresponding to different values are not mutually orthogonal. Any quantum state, pure or mixed, can be represented as the vector with $n^{2}$ coordinates given by probabilities for observation outcomes (for this state).

So, QBists demonstrated that one can ignore the problem of the interpretation of the quantum state (wave function).

The author’s intention is that in quantum-like modeling one can use the QBism methodology, the personal experience viewpoint on probability. At the same time, the notion of the personal belief state has to be used as the seed for generation of subjective probabilities.
